# Combination of FOLFOXIRI Drugs with Oncolytic Coxsackie B3 Virus PD-H Synergistically Induces Oncolysis in the Refractory Colorectal Cancer Cell Line Colo320

**DOI:** 10.3390/ijms25115618

**Published:** 2024-05-22

**Authors:** Maxim Girod, Anja Geisler, Luisa Hinze, Leslie Elsner, Babette Dieringer, Antje Beling, Jens Kurreck, Henry Fechner

**Affiliations:** 1 Department of Applied Biochemistry, Institute of Biotechnology, Technische Universität Berlin, 10623 Berlin, Germany; 2 Institute of Biochemistry, Charité—Universitätsmedizin Berlin, Corporate Member of Freie Universität Berlin and Humboldt-Universität zu Berlin, 10117 Berlin, Germany

**Keywords:** colorectal cancer, oncolytic virus, coxsackievirus, chemotherapy, combination therapy

## Abstract

FOLFOXIRI chemotherapy is a first-line therapy for advanced or metastatic colorectal cancer (CRC), yet its therapeutic efficacy remains limited. Immunostimulatory therapies like oncolytic viruses can complement chemotherapies by fostering the infiltration of the tumor by immune cells and enhancing drug cytotoxicity. In this study, we explored the effect of combining the FOLFOXIRI chemotherapeutic agents with the oncolytic coxsackievirus B3 (CVB3) PD-H in the CRC cell line Colo320. Additionally, we examined the impact of the drugs on the expression of microRNAs (miRs), which could be used to increase the safety of oncolytic CVB3 containing corresponding miR target sites (miR-TS). The measurement of cytotoxic activity using the Chou–Talalay combination index approach revealed that PD-H synergistically enhanced the cytotoxic activity of oxaliplatin (OX), 5-fluorouracil (5-FU) and SN-38. PD-H replication was not affected by OX and SN-38 but inhibited by high concentrations of 5-FU. MiR expression levels were not or only slightly elevated by the drugs or with drug/PD-H combinations on Colo320 cells. Moreover, the drug treatment did not increase the mutation rate of the miR-TS inserted into the PD-H genome. The results demonstrate that the combination of FOLFOXIRI drugs and PD-H may be a promising approach to enhance the therapeutic effect of FOLFOXIRI therapy in CRC.

## 1. Introduction

CRC accounted for approximately 9.4% of the cancer-related deaths in the year 2020, making it the second most deadly and third most diagnosed cancer type globally [1]. Though it has a relatively high chance for survival of 90% when diagnosed in stage I and II, the chance for survival drops dramatically when diagnosed in stage III and IV, to 20% [2]. A drop in fatal cases of CRC has been documented in the United States in recent years. This decline can largely be attributed to the expansion of population-based screening programs and improved identification of high-risk groups through a better understanding of the underlying mechanisms that lead to CRC development [3]. Nevertheless, projection for the 10 countries with the highest incidence of CRC suggest an increase in cases until the year 2040 [4]. For irresectable late-stage CRC, the most promising clinically approved chemotherapy regimen is the FOLFOXIRI scheme, a therapeutic schedule combining 5-FU, folinic acid (FA), Irinotecan (IRI) and OX [5]. 5-FU is a pyrimidine antagonist. The antitumor activity of 5-FU is driven by three active metabolites that cause RNA and DNA damage leading to tumor cell death. The 5-FU metabolite FdUMP irreversibly inhibits the thymidylate synthase (TS). This prevents the conversion of deoxyuridine monophosphate to deoxythymidine monophosphate (dTMP) and results in an imbalance of the deoxynucleotide pool, which leads to the arrest of cellular de novo DNA synthesis and repair. The 5-FU metabolites FUTP and FdUTP can be misincorporated into the RNA and DNA, where they can induce RNA and DNA damage, respectively [6]. IRI is a Topoisomerase I inhibitor. Its active metabolite SN-38 interacts with the Topoisomerase–DNA complex, forming a Topoisomerase Ⅰ-Irinotecan/SN-38-DNA ternary complex, which inhibits the process of DNA replication in tumor cells by inducing DNA double-stand break formations [7]. This, in turn, triggers a DNA damage response signaling cascade, leading to the induction of TP53-induced apoptosis of the cancer cells [8]. The main target of OX is the DNA, where the platinum atoms of OX form a Pt-DNA intrastrand crosslink that affects DNA replication and transcription and finally leads to the apoptosis and death of the tumor cell [9]. FA is almost consistently clinically applied in combination with 5-FU. While it does not lead to any cytotoxic effects on cancer cells itself, it synergistically increases the effect induced by 5-FU through facilitating the binding of the FdUMP to the TS complex [10]. Several phase III trials have compared the efficacy of FOLFOXIRI chemotherapy with alternative drug regimens such as FOLFIRI or FOLFOX, which involve the chemotherapeutic agents 5-FU/IRI and FA or 5-FU/OX and FA, respectively. These comparisons have consistently shown a significant benefit of the FOLFOXIRI chemotherapy in terms of progression-free survival, overall survival and response rate [11]. The FOLFOXIRI combined regimen has also exhibited synergistic effects in in vitro models, suggesting that dose reduction could potentially achieve comparable efficacy with fewer side effects when all four drugs are administered in combination [10]. Yet, in metastatic CRC, the 5-year survival rate is only around 10% with chemotherapy, which underscores its limited curative potential [12,13]. The development of drug resistance in tumor cells, which occurs with virtually all cytostatic drugs, is a primary factor contributing to the failure of chemotherapy.

Oncolytic virotherapy has emerged as a highly effective strategy in combating cancer. This approach exploits the ability of oncolytic viruses to selectively replicate in tumor cells and effectively lyse them. Through the release of damage-associated molecular patterns, pathogen-associated molecular patterns, tumor-specific and tumor-associated antigens, as well as neoantigens, this ultimately leads to the induction of a strong activation of a systemic anti-tumor immune response and tumor destruction [14]. CVB3, a single (+)-stranded RNA virus from the picornavirus family, is a new oncolytic virus that has shown potent oncolytic efficacy in different in vivo models of lung, colorectal, breast and endometrial cancer [15,16,17,18,19,20,21]. Through its efficient replication in a broad range of cancer cells, the generation of a large amount of progeny within the tumor cells and its short replication cycle of 6 to 8 h, it has an outstanding oncolytic potential [22]. Oncolytic CVB3 enters tumor cells through interaction with the coxsackievirus adenovirus receptor (CAR). However, laboratory-derived oncolytic CVB3 strains, referred as PD and PD-H, can use N- and 6-O-sulfated heparan sulfates in addition to CAR to infect tumor cells [23,24]. Consequently, the viruses exhibit a wider spectrum of tumor cell tropism, enabling the infection of CAR-deficient or CAR-low-expressing tumor cells [16]. Oncolytic CVB3 can induce side effects. This was observed in both immunocompromised and immunocompetent mice, where infection, inflammation and damage of the pancreas and the heart, representing the most susceptible organs to CVB3 in adult mice, were documented [16,20,24,25]. However, the infection of normal murine organs with oncolytic CVB3 can be prevented by equipping the viral genome with miR-TS of tissue-specific-expressed or tumor suppressor microRNAs [20,24,25,26,27]. By the binding of these miRs to their corresponding miR-TS, the viral genomic RNA is degraded in normal organs. However, in tumor cells, in which these miRs are very low or not expressed, viral replication remains unaffected, resulting in selective destruction of tumor cells [28].

In this study, we show that PD-H leads to a synergistic enhancement of FOLFOXIRI-induced oncolysis in the CRC cell line Colo320. In addition, the FOLFOXIRI drugs have little or no effect on the expression of specific miRs, which is important for ensuring the safety and efficacy of PD-H with miR-TS.

## 2. Results

### 2.1. Oncolytic Activity of CVB3 PD-H across Different Colorectal Cancer Cell Lines

**Figure 1:**
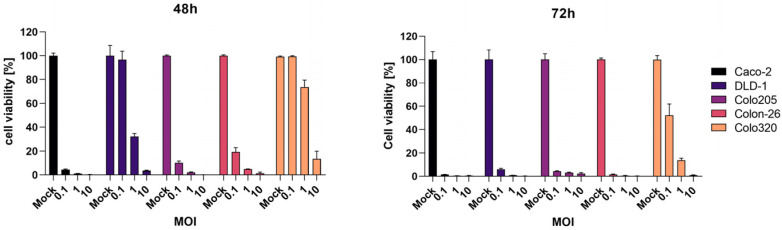
PD-H-induced cell lysis on different CRC cell lines. The CRC cell lines were infected with indicated MOIs of PD-H. Cell viability was measured by an XTT assay 48 and 72 h post infection and set relative to not-infected cells (Mock). Shown are the mean values ± SEM.

We initially assessed five colorectal cancer cell lines to determine their susceptibility to PD-H. Our aim was to identify a cell line refractory to PD-H, as investigations in such a cell line facilitate the detection of potential inhibitory, additive or synergistic effects induced by simultaneously administered agents. For this reason, Caco-2, DLD-1, Colo205, Colon-26 and Colo320 were infected with 0.1, 1 and 10 MOI of PD-H, and cell viability was determined 48 h and 72 h later. Caco-2, Colon-26 and Colo205 exhibited high susceptibility to PD-H, as evidenced by nearly complete cell lysis 48 h after PD-H infection at the lowest-used MOI of 0.1. DLD-1 cells were less sensitive to PD-H. However, almost all cells were lysed at an MOI of 0.1 72 h after infection with PD-H. In contrast, despite a tenfold-higher virus dose (MOI of 1), Colo320 cells were not completely lysed 72 h after infection, demonstrating that Colo320 cells were refractory to PD-H (Figure 1). Consequently, we selected Colo320 cells for further investigation.

### 2.2. Sensitivity of Colo320 Cells to FOLFOXIRI Drugs and Determination of the IC25 and IC50 of OX, SN-38, 5-FU, 5-FU/FA and the IC50 Equivalent of PD-H in Colo320 Cells

**Figure 2:**
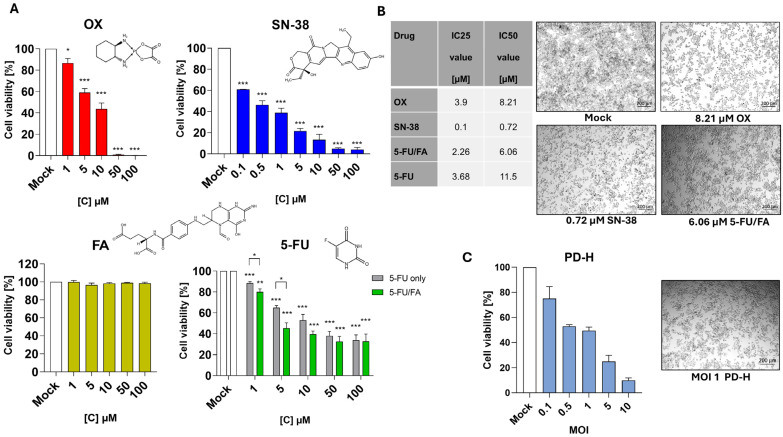
Colo320 dose–effect relation of FOLFOXIRI drugs and PD-H. (**A**) Cell viability of Colo320 cells after treatment with FOLFOXIRI drugs. Colo320 cells were treated with various drugs from the FOLFOXIRI regimen in a concentration range of 1 to 100 μM for OX, 5-FU, FA and 5-FU/FA mixture and in a concentration range of 0.1 to 100 µM for SN-38. The cell survival was measured with XTT assay 72 h after drug application. Shown are the mean values ± SEM from 3 independent experiments. Significance, * *p* < 0.05, ** *p* < 0.01, *** *p* < 0.001 compared to Mock or 5-FU vs. 5-FU/FA. Mock, untreated cells. The structural formulars for each drug have been drawn using the Thermo Scientific chemical structure search tool. (**B**) IC25 and IC50 values of FOLFOXIRI drugs and cell toxicity of the drugs at IC50. Left table, IC25 and IC50 values were calculated from measurements shown under (**A**) for each drug. Right images, Brightfield microscopy images of Colo320 cells were treated with the IC50 values of each drug 72 h after application. (**C**) Susceptibility of Colo320 cells to PD-H. Left diagram, Colo320 cells were treated with PD-H with an MOI of 0.1 to 10. Cell viability was measured with XTT assay 72 h later. Right image, Colo320 cells were treated with 1 MOI PD-H. The image was taken 72 h after infection.

To ascertain whether the combination of PD-H with the drugs of the FOLFOXIRI regimen results in synergistic, additive or inhibitory effects, we employed the Chou–Talalay constant ratio approach [29]. A recommended strategy for employing this method involves using a dose in the combination approach that is equal to or less than the IC50 for each active substance. To assess the sensitivity of Colo320 cells to the FOLFOXIRI drugs and to determine their IC50 values, a dose–response correlation was determined as part of the cytotoxicity assessment. Colo320 cells were subjected to treatment with varied concentrations of the FOLFOXIRI drugs 5-FU, FA and OX, ranging from 1 to 100 µM, and with SN-38, an active metabolite of IRI, ranging from 0.1 to 100 µM, over a 72 h investigation period. OX displayed cytotoxic effects on the cells across the whole range of concentrations, leading to the death of almost all cells at its highest concentrations of 50 and 100 µM. Lower concentrations led to above 80% cell viability at 1 µM, 58% cell viability at 5 µM and 43% cell viability at 10 µM compared to the untreated control. SN-38 showed a strong cytotoxic effect on the cells, which at concentrations of up to 10 µM was distinctly stronger than that of OX. At the highest concentrations used, 50 and 100 µM, SN-38 reduced the number of viable cells to 4% compared to an untreated control, which was very similar to OX (Figure 2A). 5-FU induced a cytotoxic effect across the entire range of measured concentrations. Especially at higher doses, the cytotoxicity of the drug was lower than that of OX and SN-38. Even at a dose of 100 µM 5-FU, cell viability was still 33% compared to the untreated control, which was significantly higher than the values determined for OX and SN-38. As expected, FA showed no cytotoxicity to the cells over the entire range of concentrations tested. Clinically, FA is always combined with 5-FU to stabilize the TS–5-FU complex, to increase the 5-FU mediated inhibition on the thymidine synthesis within the cells [10]. Hence, we assessed the cytotoxicity of 5-FU/FA using a clinically relevant drug ratio of 20:1. The application of 5-FU/FA to Colo320 cells yielded comparable maximal cytotoxicity compared to 5-FU alone, as evidenced by a maximal Colo320 cell viability of 32% at the highest-used 5-FU concentration of 100 µM. However, a more potent cytotoxicity was achieved at lower 5-FU concentrations (1 µM and 5 µM) in the 5-FU/FA approach when compared to 5-FU alone, indicating the positive effect of FA to 5-FU induced cytotoxicity. 

Utilizing the dose–response relationships, the IC25 and IC50 values of the FOLFOXIRI drugs were calculated as follows: 3.9/8.21 µM for OX, 0.1/0.72 µM for SN-38, 3.68/11.5 µM for 5-FU and 2.26/6.06 µM for 5-FU/FA (Figure 2B). Based on cell culture images taken 72 h after treatment, it is evident that the calculated IC50 of OX, SN-38 and 5-FU/FA induced a comparable cytotoxic effect in Colo320 cells (Figure 2B).

To assess the sensitivity of Colo320 cells to PD-H, the cells were infected with 0.1 to 10 MOI of the virus for 72 h. Cell viability was reduced to 80% at the lowest virus dose of 0.1 MOI and decreased linearly to only 10% at the highest-used dose of 10 MOI compared to the untreated control. When using 1 MOI of PD-H, a decrease in cell viability of about 50% was observed (Figure 2C). This dose was determined as the viral equivalent of the IC50 for the subsequent combination approaches.

### 2.3. PD-H Synergistically Enhances Oncolysis of OX, SN-38 and 5-FU/FA

**Figure 3:**
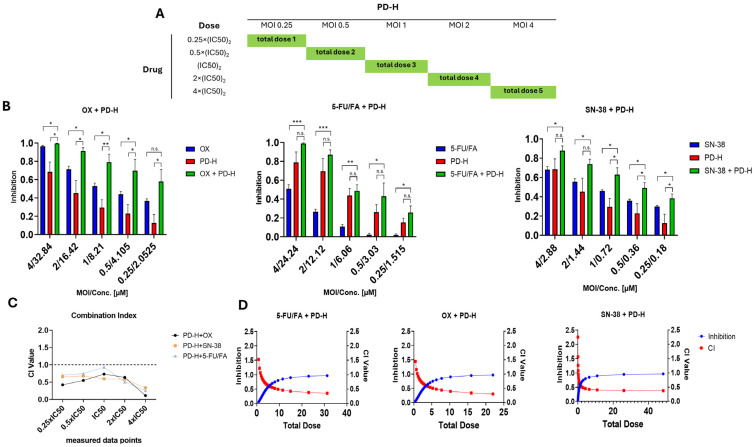
Cell growth inhibition upon combination of OX, SN-38 and 5-FU/FA with PD-H. (**A**) Application scheme. Shown is the diagonal constant ratio scheme and the resulting data points used for measurement. (**B**) Cell growth inhibition. Colo320 cells were infected with PD-H for 1 h and treated thereafter with FOLFOXIRI drugs or the cells treated with PD-H or FOLFOXIRI drugs alone. Cell viability was measured by XTT assay 72 h after treatment. Inhibition is shown as 1-% of viable cells. Shown are the mean values ± SEM from 3 independent experiments. Significances, * *p* < 0.05, ** *p* < 0.01, *** *p* < 0.001. n.s., not significant. (**C**) Combination Index. The CI value for each measured data point is shown for each combination. CI values below the threshold of 1 indicate a synergic interaction, whereas values of 1 indicate additive effects, and values above 1 indicate inhibitory effects. (**D**) Simulated dose–effect curves. Dose–effect curves with the corresponding CI values were generated by the CompuSyn software (version 1.0) based on the measured data points from (**B**). The total dose is defined as the sum of the viral MOI and the drugs’ μM concentrations. An inhibition of 1 therefore corresponds to 100% cell death, and an inhibition of 0.5 corresponds to 50% cell death.

To determine whether PD-H synergically, additively, or antagonistically complements the oncolysis of OX, SN-38 and 5-FU/FA, we employed the diagonal constant ratio design proposed by Chou and Talalay [29]. This specific approach allows the full utilization of the CompuSyn software (version 1.0) to calculate a CI for each measured data point and simulate a meaningful dose–effect curve across the full spectrum of inhibition for each combination [30]. Colo320 cells were treated with the FOLFOXIRI drugs and PD-H alone, or with each respective combination, according to the scheme shown in Figure 3A, and the inhibition of cell growth was determined relative to the untreated control 72 h later. 

In all investigations, the inhibition exerted by the combinations of PD-H with the drugs outperformed the inhibition of each single agent in every measured data point. The combination of OX with PD-H showed the most substantial increase in inhibition of cell growth. At the lowest-used combined drug/virus concentration, it reached 57% inhibition, which was distinctly higher than the 37% and 12% determined after OX and PD-H application alone, respectively. At the highest-used drug/virus combination, cell growth was completely inhibited, whereas OX and PD-H application alone led to inhibition of 96% (OX) and 68% (PD-H), each compared to an untreated control, respectively. Moreover, compared to the individual application of OX and PD-H, for each used dose, the growth inhibition was significantly higher in the OX/PD-H combination approach. A similar outcome was achieved for the combination of SN-38 with PD-H. The combination led to 38% growth inhibition at the lowest concentration compared to 30% (SN-38) and 12% (PD-H) and, at the highest concentration, to 88% compared to 68% each for SN-38 and PD-H. The combination of drug and virus exhibited significantly higher efficacy at each dose level compared to either the virus or the drug administered alone. Taking into consideration that 5-FU and FA are utilized together in the clinical setting, we did not investigate PD-H in combination with 5-FU but only with 5-FU/FA. At the lowest-used concentration, the combination of 5-FU/FA with PD-H resulted in 25% inhibition of cell growth compared to 1.8% (5-FU/FA) and 15% (PD-H), respectively. At the highest-used concentration, the combination led to an inhibition of cell growth of 99% compared to 51% for 5-FU/FA and 79% for PD-H. At all the concentrations used, the inhibition of cell growth determined for the combination of 5-FU/FA/PD-H was significantly higher than that of 5-FU/FA, while there was no significant difference compared to PD-H (Figure 3B). 

To characterize the combination approaches regarding synergism, additivity or antagonism based on the cell growth inhibition data, we determined a CI for each measured data point. A CI below the value of 1 suggests synergic effects between two drugs, whereas a value between 0.9–1.1 suggests additive interactions. At a CI above 1, the combination can be considered as antagonistic. Almost all the measured data points of the three combinations were below the value 0.9, indicating synergic interactions of OX, SN-38 and 5-FU/FA with PD-H (Figure 3C). As an exception, the combination of 5-FU/FA with PD-H only led to a CI just below 1 at IC50, which leads to the assumption that, at this concentration, there was a strong additive and not a synergistic interaction for this combination therapy. 

Generally, a lower CI can be associated with more potent synergism. Thus, we detected for the three drug/PD-H combinations the strongest synergism at the highest used concentrations of the individual agents (Figure 3C). The diagonal constant ratio approach allows us to simulate the dose–effect curve of each agent. Therefore, we proceeded to plot the inhibition projected to be exerted on the cells and its corresponding CI value. From the simulated curves, it becomes visible that every combination is projected to have a direct dose–CI correlation, as, for all three combinations, the CI value decreases with the increasing total dose (Figure 3D). 

Additionally, the simulation allowed us to calculate the concentration reduction of each chemotherapeutic drug needed to reach 50% inhibition on the cell growth and to compare it to the IC50 value of each drug alone. The simulation revealed that to attain a 50% inhibition, the calculated concentrations of OX and PD-H in the combined approach were 2.42 µM and 0.295 MOI, respectively. Similarly, for the combination of SN-38 and PD-H, the calculation yielded 0.36 µM and 0.49 MOI, while for 5-FU/FA and PD-H, it resulted in 3.617 µM and 0.6 MOI, respectively. Consequently, it can be assumed that the dose used in the OX/PD-H, SN-38/PD-H and 5-FU/FA/PD-H combinations to inhibit cell growth can be reduced 3.4-fold, 2-fold and 1.7-fold to reach the same potency as each agent alone. 

In summary, these results show that PD-H interacts synergistically with the drugs of the FOLFOXIRI chemotherapy regimen over a broad dose range.

### 2.4. 5-FU/FA Reduces PD-H Replication but Only at High Concentrations

**Figure 4:**
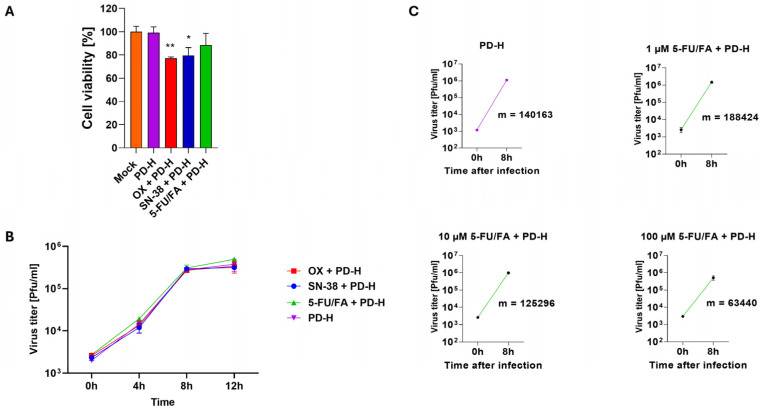
Influence of chemotherapeutics on replication of PD-H. (**A**) Cell viability in the FOLFOXIRI/PD-H combination approach. Colo320 cells were infected with PD-H at an MOI of 1 or were infected with PD-H for 1 h and thereafter incubated with the IC50 of the FOLFOXIRI drugs. Cell viability was measured by the XTT assay 12 h later. Significances, * *p* < 0.05, ** *p* < 0.01 vs. PD-H. Mock, untreated cells. (**B**) Viral replication curve for combinations of FOLFOXIRI drugs with PD-H or with PD-H alone. Colo320 cells were infected as described with FOLFOXIRI drugs and PD-H under (**A**). Viral titers were determined by plaque assay 0, 4, 8 and 12 h post infection. (**C**) The slopes (m) of the mean rates of change between the primary uptake and the final titers 8 h post-infection are displayed.

Viral replication plays an essential role in cancer therapy with oncolytic viruses, as the virus-induced destruction of tumor cells and the spread of the virus in the tumor microenvironment are crucial for the induction of an antitumor immune response and therapeutic success. We therefore investigated the influence of OX, SN-38 and 5-FU/FA on the replication of PD-H in Colo320 cells to find out whether the drugs inhibit the viral replication. The cells were treated with 1 MOI PD-H for 1 h and incubated without (control) or with the IC50 of the FOLFOXIRI drugs. To mitigate potential secondary inhibitory effects resulting from the cytotoxicity of the drugs, the temporal scope of the viral replication study was confined to a 12 h window. This limitation was based on the recognizable manifestation of a modest cytotoxic effect observed in Colo320 cells following treatment with the drug/PD-H combinations at this specific time point (Figure 4A). The viral virus growth curve showed similar virus growth over the entire study period for the control samples treated with PD-H and the samples co-treated with the drugs and PD-H. This indicates that the drugs have no effect on the replication of PD-H (Figure 4B). However, for 5-FU/FA, these data were surprising, as it has been shown that 5-FU can directly inhibit the replication of RNA viruses due to its metabolite 5-FUTP, which functions as a substrate for the viral RNA-dependent RNA polymerase [31]. We therefore conducted an additional experiment with 5-FU/FA, in which we used a broad dose spectrum of 5-FU between 1 µM and 100 µM. 5-FU/FA was administered to Colo320 cells 24 h prior to PD-H infection. To mitigate potential secondary inhibitory effects of 5-FU/FA treatment on virus replication, the determination of virus replication was restricted to 8 h, corresponding to the time necessary for one replication cycle of PD-H to complete. High concentrations of 5-FU/FA of 100 µM inhibited the replication of PD-H, as demonstrated by a 2.2-fold decrease in the mean rate of change of the equation between the primary uptake and the final virus titers compared to untreated PD-H-infected controls. Conversely, the mean doses of 10 µM exhibited no discernible effect on PD-H replication, whereas, at a low dose of 1 µM for 5-FU/FA, there was a slight increase. The latter could be attributed to the scenario where, at very low concentrations, the positive impact of FA on cell metabolism outweighs the negative effects associated with its influence on the activity of 5-FU (Figure 4C). 

These results indicate that SN-38 and OX have no direct effect on PD-H replication, whereas 5-FU/FA inhibits PD-H replication in Colo320 cells, but only at very high doses.

### 2.5. FOLFOXIRI Drug Does Not Increase miRs Expression in Colo320 Cells and Does Not Affect Stability of miR-TS Inserted into the Genome of PD-H 

**Figure 5:**
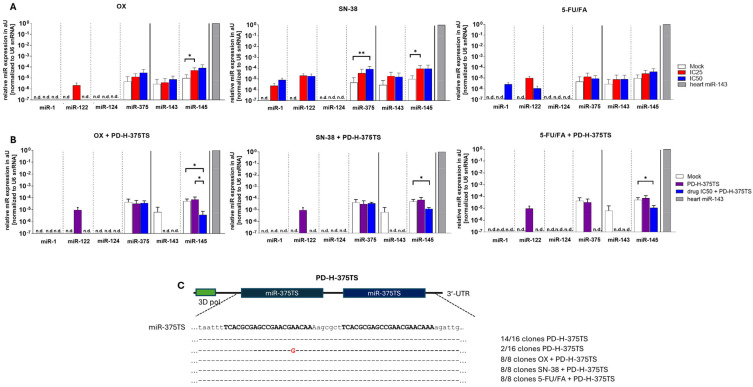
Relative expression level of tissue-specific-expressed miR-1, miR-122, miR-124, miR-375 and tumor-suppressor miR-143 and miR-145 in Colo320 cells after treatment with FOLFOXIRI drugs and PD-H-375TS. (**A**) MiR expression in Colo320 cells after application FOLFOXIRI drugs. Colo320 cells were incubated with the IC25 or IC50 of the FOLFOXIRI drugs. MiR expression was determined by qRT-PCR and normalized against the expression levels measured for endogenous U6 snRNA. MiR expression values are shown relative to the expression of the miR-143 in mouse heart (set as 1). The data represent means ± SEM of 3 independent experiments, each in triplicate. n.d., not detected; aU, arbitrary units; significance, * *p* < 0.05, ** *p* < 0.01. (**B**) MiR expression in Colo320 cells after combining FOLFOXIRI drugs with PD-H-375TS. Colo320 cells were infected with PD-H-375TS at an MOI of 1 for 1 h and treated with the IC50 of FOLFOXIRI drugs, or the cells were treated with PD-H-375TS at an MOI of 1 alone. MiR expression was determined by qRT-PCR, as described under (**A**), 48 h after viral infection. The data represent means ± SEM of 3 independent experiments, each in triplicate. n.d., not detected; aU, arbitrary units; significance, * *p* < 0.05. (**C**) Sequence alignment of the miR-375TS. Colo320 cells were treated with FOLFOXIRI drugs and PD-H-375TS or PD-H-375 alone, as described under (**B**). Viral RNA was isolated 48 h after infection, and the miR-375TS was cloned and sequenced.

To increase its safety, oncolytic CVB3 can be engineered with the miR-TS of tissue-specific or tumor suppressor miRs, thereby impeding viral replication in normal tissue while allowing replication in tumor cells continue unaffected [20,24,25,26,27]. A critical prerequisite for the success of this approach is the high expression of the miRs in normal organs, juxtaposed with their low expression or absence in tumor target cells [28]. However, chemotherapy can increase miR expression in cancer cells [32,33,34,35,36], and, therefore, the use of FOLFOXIRI drugs could potentially lead to an undesirable reduction in viral replication of miR-TS-containing oncolytic CVB3 in CRC cells. The heart, liver, brain and pancreas are important target organs of CVB3 in humans, and miR-1, miR-122, miR-124 and miR-375 are highly expressed in the heart (miR-1), in the liver (miR-122), in the brain (miR124) and in the pancreas (miR-375) [37]. Therefore, we investigated the expression of these miRs in Colo320 cells. In addition, two tumor-suppressor miRs (miR-143 and miR-145) were also investigated, as they have already been used in oncolytic CVB3 [27]. Colo320 cells were treated with the IC50 and IC25 of OX, SN-38 and 5-FU/FA for 48 h, and miR expression was determined by quantitative (q)RT-PCR. The six miRs examined were not expressed in the Colo320 cells or their basal expression was very low, as shown by a comparison with the expression of miR-143 in mouse heart tissue, in which the miR-143 is strongly expressed. Regardless of the FOLFOXIRI drug used, there was either no or only a slight increase in the levels of the miRs compared to the mock control (Figure 5A). A similar result was obtained when Colo320 cells were treated with the FOLFOXIRI drugs at an IC50 and co-infected with 1 MOI of the PD-H derivate PD-H-375TS, containing miR-375TS in the 3′UTR of the viral genome. No or only a slight increase in the expression of the six miRs was observed (Figure 5B). Using the latter approach, we also examined whether the FOLFOXIRI drugs affect the stability of miR-375TS in PD-H-375TS. Cloning of miR-375TS from the samples treated with FOLFOXIRI drugs and PD-H-375 and from a control that had been infected with PD-H-375TS but was not treated with FOLFOXIRI drugs revealed that, in both, no deletion of miR-375TS occurred in the PD-H genome. Among the 16 miR-375TS sequences cloned from the PD-H-375TS control group, one nucleotide substitution was detected in two clones, while the other 14 clones completely matched the original miR-375TS sequence. In addition, no mutations within miR-375TS were detected in any of the eight miR-TS clones from the FOLFOXIRI drug/PD-H-375TS combination approach (Figure 5C).

In summary, these results show that treatment with FOLFOXIRI drugs does not or only slightly increases the expression of the selected miRs in Colo320 cells and does not decrease the stability of miR-TS.

## 3. Discussion

The treatment landscape of colorectal carcinoma (CRC) has evolved over several decades, with various chemotherapeutic agents playing pivotal roles. Among these therapies, 5-FU has traditionally been one of the most widely used drugs. In the past decades, new drugs have been developed to enhance the inhibition of cell proliferation and tumor growth in CRC, including cisplatin-based agents and topoisomerase I inhibitors [38]. However, fast-developing resistances and incomplete eradication of tumor cells remain two of the biggest challenges when using these chemotherapeutic agents as monotherapies [6,39,40]. Therefore, combination therapies are now first-line therapies for the treatment of CRC. In this context, the FOLFOXIRI regimen has become increasingly important, as it is superior to other combination therapies with chemotherapeutic agents in terms of progression-free survival, overall survival and response rate [11,41]. However, despite better results, the therapeutic success of FOLFOXIRI chemotherapy in advanced or metastatic CRC is still unsatisfactory. Therefore, the combination of standard chemotherapies and other tumor-targeted therapies has gained increasing interest [42]. 

In this study, we have examined the synergic, additive, and antagonistic characteristics of a potential combination approach including the drugs of the FOLFOXIRI scheme and the oncolytic CVB3 PD-H on a cellular level in the CRC cell line Colo320. The Colo320 cell line has a neuroendocrine origin characterized by the production of serotonin, noradrenaline, epinephrine, adrenocorticotropic hormone and parathyroid hormone [43]. In addition, its molecular phenotype is distinctly different from that of other human CRC cell lines [44]. This could be one reason why only this cell line was refractory to PD-H, which qualified it for use in this study. We show that the drugs utilized in FOLFOXIRI chemotherapy synergize with PD-H, resulting in significantly heightened cytotoxicity of the combination approach compared to using either the drugs or the virus alone. This effect was observed across all three FOLFOXIRI chemotherapeutic agents, OX, SN-38 and 5-FU, and remained detectable across a broad dose range. Furthermore, the viral replication was unaffected by OX and SN-38 and only marginally reduced when high concentrations of 5-FU were employed.

To achieve the best therapeutic results, the different cancer therapeutics used in combination should act additively or, even better, synergistically. The concept of synergism strongly relies on the increase of potency based on interactions between the different therapeutic strategies used. Although the elucidation of these mechanisms is still resource and time consuming, the determination of the synergistic, additive or antagonistic effects of a combined treatment was made possible in a simple way by the development of a mathematical prediction model by Chou and Talalay [45]. Using this method, we determined that OX and SN-38 synergize with PD-H, while 5-FU is additive or synergistic with PD-H, depending on the used dose. In multimodal therapies, the timing of the application of each active agent plays a vital role. A typical FOLFOXIRI cycle in patients consists of the administration of OX and Leucoverin for 2 h, followed by 30 to 60 min of IRI, and finishing with 48 h of 5-FU [46], which raises the question of when to use an oncolytic virus to ensure the best efficacy as part of combination therapy. To take advantage of the synergistic effect between the FOLFOXIRI drugs and PD-H, it could be beneficial to apply the virus 24 to 48 h before the start of the drug cycle. Since a replication cycle of CVB3 is approximately 6 to 8 h, delayed administration of the FOLFOXIRI drugs could allow the virus to replicate and spread in the tumor cells before the drugs cause toxicity to the tumor cells and slow viral spread. Moreover, the viral spread among tumor cells would increase the potential for drug–virus interactions within tumor cells. Another potential consequence of the synergistic activity of FOLFOXIRI drugs and PD-H is the possibility of reducing the dose of the single agent in the combination approach. The latter may be of considerable importance for colorectal cancer patients, as treatment with FOLFOXIRI is very stressful for patients and is therefore only an option for patients who are in sufficiently good physical condition to tolerate the side effects [11,47]. Lowering the dose of the drugs, especially of IRI, which constitutes a primary determinant of the exacerbated side effects observed in FOLFOXIRI chemotherapy, holds promise for attenuating adverse effects and may thereby confer benefits to patients [48]. Moreover, by adjusting the dose of the chemotherapeutic agents, patients who were previously ineligible for FOLFOXIRI therapy due to not meeting the inclusion criteria could now become candidates for the FOLFOXIRI/PD-H combination therapy. 

The results presented in this study confirm data of earlier studies that demonstrate that chemotherapeutic drugs act additively or synergistically with oncolytic viruses in CRC cells [49,50]. Moreover, our data strongly encourage further research of the combination of the FOLFOXIRI scheme with the oncolytic virus PD-H for the treatment of CRC in vivo. If the synergistic interactions observed here in vitro can be transferred to in vivo models, the benefits of this combination are likely to extend beyond merely enhancing the direct killing by the FOLFOXIRI drugs and by PD-H. The reason for this is the ability of oncolytic CVB3 to induce immunogenic cell death in cancer cells, thereby triggering a robust systemic immune response that acts against the virus-infected tumors as well as metastases [15,24,51]. 

The safety of oncolytic viruses is a crucial factor for their use in cancer therapy. In RNA viruses such as PD-H, the incorporation of miR-TS into the viral genome has proven to be extremely effective in preventing undesirable viral replication and associated toxicity in normal tissues [22,52,53]. This is achieved by the fact that the selected miRs that correspond to the miR-TS are highly expressed in normal cells, while they are lowly expressed or absent in tumor cells [28]. However chemotherapeutic drugs can enhance the expression of miRs in cancer cells [32,33,34,35,36], which may increase the risk of undesired inhibition of viral replication in these cells if an miR that binds to a corresponding miR-TS in the viral genome is affected. Here, we investigated the expression of the four tissue-specific-expressed miRs (miR-1, miR-122, miR-124, miR-375) and two tumor-suppressor miRs (miR-143 and miR-145) in Colo320 cells following treatment of the cells with chemotherapeutic agents and PD-H. These miRs were chosen because they are highly expressed in CVB3 target tissues in humans (heart, liver, brain and pancreas) but minimally expressed in colorectal carcinoma cell lines. Therefore, equipping the viral genome with corresponding miR-TS may be suitable to prevent CVB3 replication in these organs but not in colorectal carcinoma cells. Starting from a very low basal expression level, the FOLFOXIRI drugs do not or only slightly increase the expression level of the respective miRs in Colo320 cells. Even when an increase of the miR expression was detected, the level remained at least 10,000-fold below values that can be considered as “high”. According to our experience and findings of previous studies [24,26,54], such low-expressed miRs do not hinder the replication of miR-TS-containing PD-H. Another significant observation in this context is that treatment with each FOLFOXIRI drug did not increase the mutation rate within the miR-TS. This finding is crucial as it confirms the stability of the miR-TS within the PD-H genome following FOLFOXIRI drug treatment, which is important for the safety of the virus. Nevertheless, it is imperative to note that further in vivo studies involving long-term applications will be indispensable to draw a conclusive assessment in this regard.

Recently, we demonstrated that PD-H can be easily and quickly adapted to Colo320 cells [55]. This was achieved with a newly developed protocol that combines direct viral evolution with genetic modification of the viral genome. The adapted virus overcame the resistance of Colo320 cells to PD-H, shown by a 100-fold increase in viral replication and significantly improved tumor cell lysis in vitro. In addition, the growth of Colo320 tumors in mice was effectively inhibited by the virus, which was not observed with PD-H [55]. These results demonstrate that the efficacy of oncolytic viruses can be selectively enhanced in primary resistant CRC cells, thereby opening up the possibility of using them in the context of personalized virotherapy for patients with individual therapy resistance. Moreover, concerning the co-application of chemotherapy and virotherapy, the combination of personalized virotherapy and chemotherapy will further increase therapeutic efficiency in cancer therapy and thus be beneficial for cancer patients.

In summary, we have shown here that the oncolytic CVB3 PD-H synergistically enhances the cytotoxic activity of the drugs of the FOLFOXIRI regimen in the CRC cell line Colo320. Furthermore, the chemotherapeutic agents do not affect specific properties that determine the efficacy of the virus in Colo320 cells. The combination with PD-H immunotherapy, therefore, has the potential to improve the efficacy of conventional FOLFOXIRI chemotherapy in CRC. 

## 4. Materials and Methods

### 4.1. Cell Lines

Colorectal carcinoma cell lines DLD1, Colo320, Colo205 and Colon-26 were grown in RPMI 1640 (c.c pro GmbH, Oberdorla, Germany) supplemented with 10% fetal calf serum (FCS; c.c pro GmbH), 1% penicillin-streptomycin (P/S) (Sigma-Aldrich, Taufkirchen, Germany), 1% L-glutamine and 1 mM Na-pyruvate (Sigma-Aldrich). Caco-2 cells were cultured in Dulbecco’s modified Eagle’s medium (DMEM, Th. Geyer GmbH & Co. KG, Renningen, Germany) supplemented with 10% FCS, 1% P/S, 2 mM L-glutamine and 2% sodium pyruvate. HeLa cells (human cervical carcinoma) were cultured in modified Eagle’s medium (MEM) complete medium, which consists of MEM (Life Technologies, Darmstadt, Germany) supplemented with 2 mM L-glutamine, 5% FCS, 1% NEAA (non-essential amino acids, Life Technologies, Carlsbad, Germany), 0.02 M Hepes buffer (2-(4-(2-Hydroxyethyl)-1-piperazinyl)-ethansulfonicacid, Life Technologies) and 1% P/S. CHO-K1 cells (Chinese hamster ovary cells) were cultured in DMEM medium supplemented with 10% FCS and 1% P/S.

### 4.2. Viruses

The viruses PD-H and PD-H-375TS were produced as described previously [24]. Briefly, for generation of PD-H, the plasmid pJET-CVB3-PD-H containing the cDNA of the CVB3 strain PD-H was transfected in CHO-K1 cells. For generation of PD-H-375TS, the plasmid pJET-CVB3-PD-H-375TS containing the cDNA of PD-H equipping with two miR-375TS in the 3′UTR of the viral genome was transfected into HEK-293 cells. Plasmid transfection was carried out with PEImax (Polysciences Europe GmbH, Hirschberg an der Bergstrasse, Germany) when cells reached confluence of about 70%. Then, 48 to 72 h post transfection, when cell lysis became visible, cells were subjected to 3 freeze and thaw cycles, and plaque assays were carried out to determine the virus titer. PD-H and PD-H-375TS were further propagated by infection of CHO-K1 cells to generate enough virus for the experiments. 

### 4.3. FOLFOXIRI Drugs

5-FU, OX and FA were provided from Sigma-Aldrich. SN-38 was provided from Hölzel Diagnostika Handels GmbH (Cologne, Germany). 5-FU and SN-38 were dissolved in DMSO (Carl Roth GmbH plus Co.KG, Karlsruhe, Germany), OX and FA were dissolved in ddH_2_O to generate 10 µM stock solutions. The stock solutions of 5-FU, OX and FA were stored at 4 °C. The stock solution of SN-38 was stored at −80 °C up to use. When 5-FU and FA were used together, the ratio of 5-FU/FA was 20:1. In this case only, the concentration of 5-FU is indicated. 

### 4.4. Cell Viability Assay 

Cell viability was measured using the Cell Proliferation Kit II (XTT) (Roche Diagnostics, Mannheim, Germany) according to the instructions of the manufacturer. Colo320, Colo205, Colon-26 cells were seeded into 96-well plates at 5 × 10^4^ cells per well and DLD-1 and Caco2 cells at 2 × 10^4^ cells per well. Then, 24 h later, cells were treated with viruses at concentrations of 0.1 to 10 MOI and/or chemotherapeutics at concentrations of 0.1 to 100 µM for 1 h. Absorbance levels were measured 48 h and 72 h after treatment using the TriStar2 MultimodeReader LB942 (Berthold Technologies, Bad Wildbad, Germany). As dead control, cell layers were treated with 50 µL of 5% Triton X-100 solution (Carl Roth GmbH plus Co.KG).

### 4.5. Plaque Assay

HeLa cells were seeded in 24-well cell culture plates as confluent monolayers. After 24 h, the medium was removed, and cells were overlaid with serial 10-fold dilutions of solution harvested from virus-infected cells. After incubation at 37 °C for 30 min, the supernatant was removed, and the cells were overlaid with 500 μl liquid agar containing BD-Difco Noble Agar (Thermo Fisher Scientific Inc., Waltham, MA, USA), 68% MEM (Life Technologies) and 9.1% FCS. After incubation at 37 °C for 3 days, cells were stained with 1× 3-(4,5-dimethylthiazol-2-yl)-2,5-diphenyl tetrazolium bromide iodotetrazolium chloride (MTT/INT) solution (VWR international GmbH, Darmstadt, Germany). Virus titers were determined by plaque counting 4 h after staining. 

### 4.6. Determination of IC25 and IC50 of Chemotherapeutic Agents

The IC25 and IC50 values were calculated using fit spline/LOWESS regression analysis performed in GraphPad Prism (Version 8.4.2 GraphPad Software Inc., La Jolla., CA, USA) based on data obtained in the XTT assay.

### 4.7. Determination of Combination Index-Value Using the Chou–Talalay Constant Ratio Approach

Colo320 cells were seeded in a 96-well plate at 2.4 × 10^4^ cells per well and infected 24 h later with PD-H for 1 h. Thereafter, the medium was replaced with fresh medium containing the drugs. Virus/drug was applied according to the constant ratio approach [29] as follows: 0.25 MOI/1/4× IC50, 0.5 MOI/1/2× IC50, 1 MOI/IC50, 2 MOI/2× IC50, 4 MOI/4× IC50. Cells were incubated at 37 °C for further 72 h, and cell viability was measured by XTT assay. The data were entered into the CompuSyn software (version 1.0) (ComboSyn, Inc., Paramus, NJ, USA), which subsequently calculated the combination index (CI) value for each measured data point, as well as presented a simulated calculation across the full spectrum of affected fractions [45]. Calculations with in the CompuSyn software (version 1.0) were based on the medium-effect equation and the corresponding question for CI value calculation [30].

### 4.8. Viral Growth Curve 

Colo320 cells were seeded into 96-well plates at 2.4 × 10^4^ cells per well. After 24 h, the medium was replaced by fresh medium containing 1 MOI of PD-H. After incubation for 1 h at 37 °C, the virus-containing cell supernatant was replaced by full medium containing the respective IC50 values for each chemotherapeutic drug, and the cells were further incubated. Virus was released from the cells by 2× freeze–thaw cycles 0, 4, 8, 12, 24 and 48 h after virus application. Cell debris was discarded by centrifugation and supernatant analyzed by plaque assay to determine the virus titers. 

### 4.9. Median PD-H Growth Rate Determination after 5-FU/FA Application

Colo320 cells were seeded into 96-well plates at 2.4 × 10^4^ cells per well. After 24 h, the cell culture medium was replaced, and the cells were infected with 1 MOI of PD-H for 1 h. Thereafter, the medium was replaced by fresh medium containing 5-FU/FA at concentrations of 1, 10, 100 µM. Samples were taken at 0 and 8 h post infection, and analyzed using a plaque assay, as described earlier. 

### 4.10. Determination of miR Expression by qRT-PCR

Total RNA was isolated from Colo320 cells or heart tissue of adult Balb/C mouse using TRIzol^®^ Reagent (Thermo Fisher Scientific Inc.) and EurX Universal RNA Purification Kit (Roboklon GmbH, Berlin, Germany). Fifty ng RNA was reverse transcribed using the high-capacity cDNA reverse transcription kit (Applied Biosystems, Waltham, MA, USA). Expression levels of miR-1, miR-122, miR-124, miR-143, miR-145 and miR-375 were determined by real-time PCR using the Taq-Man MicroRNA Assays hsa-miR-1, hsa-miR-122, bfl-miR-124, hsa-miR-143, hsa-miR-145 and hsa-miR-375 (Applied Biosystems) in the CFX96™ Real-Time-System (Bio-Rad Laboratories GmbH, Hercules, CA, USA) and CFX Opus 96 Real-Time PCR System (Bio-Rad Laboratories GmbH) according to the manufacturer’s instruction. U6snRNA expression was determined and used as an internal control for normalization. Only CT values below 40 were considered for analysis. The relative miR expression was determined using the ddCT calculation method. 

### 4.11. Genetic Stability of miR-TS

Colo320 cells were seeded in a 96-well plate at 2.4 × 10^4^ cells per well and infected 24 h later with PD-H-375TS for 1 h. The medium was replaced with fresh medium containing the IC50 of OX, 5-FU/FA or SN-38. After 48 h, cell supernatant was removed from the cell culture and virus was released from the cells by three freeze–thaw cycles. Viral RNA was isolated using the NucleoSpin^TM^ RNA virus kit (Macherey-Nagel, Düren, Germany) according to the manufacturer’s protocol. Viral RNA was reverse transcribed using a high-capacity cDNA reverse transcription kit (Applied Biosystems), and the genomic region of PD-H-375TS containing the miR-375TS was amplified using the forward primer IF-pUC19-For (5′-CGGTACCCGGGGATCCCTAAAAATGTTACTTGAGAAGCTT-3′), which binds upstream the miR-375TS in the region encoding for the 3D Pol, and the reverse primer IF-pUC19-R (5′-CGACTCTAGAGGATCGCGGAGAATTTACCCCTACTGTA-3′), which binds downstream the miR-375TS in the 3′UTR. Both primers were designed with the online infusion primer designing tool (Takara Bio, Shiga, Japan). The PCR fragments were cloned into the plasmid Puc19 using the In-Fusion HD Cloning Kit (Takara Bio, Shiga, Japan) according to manufacturer’s instructions. Plasmids were isolated with NucleoSpin™ Plasmid EasyPure (Macherey-Nagel), and the miR-TS were sequenced by LGC Genomics GmbH (Berlin, Germany).

### 4.12. Statistical Analysis

Statistical analysis was performed with Graph-Pad Prism 8.4.2 (GraphPad Software, Inc., La Jolla, CA, USA). Results are expressed as the mean ± SEM. Statistical significance was determined by use of the two-tailed unpaired Student *t*-test was used for all analysis concerning the expression of miRs. For all other analyses, a one-tailed homoscedastic *t*-test was used. Differences were considered significant at *p* < 0.05.

## Data Availability

Data will be supplied following reasonable requests.
